# Runtime and aPTT predict venous thrombosis and thromboembolism in patients on extracorporeal membrane oxygenation: a retrospective analysis

**DOI:** 10.1186/s13613-016-0172-2

**Published:** 2016-07-19

**Authors:** Franziska C. Trudzinski, Peter Minko, Daniel Rapp, Sebastian Fähndrich, Hendrik Haake, Myriam Haab, Rainer M. Bohle, Monika Flaig, Franziska Kaestner, Robert Bals, Heinrike Wilkens, Ralf M. Muellenbach, Andreas Link, Heinrich V. Groesdonk, Christian Lensch, Frank Langer, Philipp M. Lepper

**Affiliations:** Department of Internal Medicine V - Pneumology and Critical Care Medicine, University Hospital of Saarland, Homburg, Germany; Department of Diagnostic and Interventional Radiology, University Hospital of Saarland, Homburg, Germany; Institutes for Medical Biometry, Epidemiology and Medical Informatics, Saarland University, Homburg, Germany; Department of Cardiology, Kliniken Maria-Hilf GmbH, Mönchengladbach, Germany; Department of Pathology, University Hospital of Saarland, Homburg, Germany; Department of Anaesthesiology and Intensive Care Medicine, University Hospital of Würzburg, Würzburg, Germany; Department of Internal Medicine III – Cardiology, and Critical Care Medicine, University Hospital of Saarland, Homburg, Germany; Department of Anaesthesiology, Critical Care Medicine and Pain Therapy, University Hospital of Saarland, Homburg, Germany; Department of Thoracic and Cardiovascular Surgery, University Hospital of Saarland, Homburg, Germany

## Abstract

**Background:**

Even though bleeding and thromboembolic events are major complications of extracorporeal membrane oxygenation (ECMO), data on the incidence of venous thrombosis (VT) and thromboembolism (VTE) under ECMO are scarce. This study analyzes the incidence and predictors of VTE in patients treated with ECMO due to respiratory failure.

**Methods:**

Retrospective analysis of patients treated on ECMO in our center from 04/2010 to 11/2015. Patients with thromboembolic events prior to admission were excluded. Diagnosis was made by imaging in survivors and postmortem examination in deceased patients.

**Results:**

Out of 102 screened cases, 42 survivors and 21 autopsy cases [mean age 46.0 ± 14.4 years; 37 (58.7 %) males] fulfilling the above-mentioned criteria were included. Thirty-four patients (54.0 %) underwent ECMO therapy due to ARDS, and 29 patients (46.0 %) with chronic organ failure were bridged to lung transplantation. Despite systemic anticoagulation at a mean PTT of 50.6 ± 12.8 s, [VT/VTE 47.0 ± 12.3 s and no VT/VTE 53.63 ± 12.51 s (*p* = 0.037)], VT and/or VTE was observed in 29 cases (46.1 %). The rate of V. cava thrombosis was 15/29 (51.7 %). Diagnosis of pulmonary embolism prevailed in deceased patients [5/21 (23.8 %) vs. 2/42 (4.8 %) (*p* = 0.036)]. In a multivariable analysis, only aPTT and time on ECMO predicted VT/VTE. There was no difference in the incidence of clinically diagnosed VT in ECMO survivors and autopsy findings.

**Conclusions:**

Venous thrombosis and thromboembolism following ECMO therapy are frequent. Quality of anticoagulation and ECMO runtime predicted thromboembolic events.

## Background

In 1855, Rudolf Virchow established a theory on coagulation [1]. His triad of causes for thrombogenesis consisting of increased blood viscosity, altered blood flow and endothelial injury is still valid [2]. When transferring Virchow’s triad to patients on extracorporeal membrane oxygenation (ECMO), they are at high risk of thrombosis and thromboembolism. Pump-induced platelet activation as well as inflammation and consumption of clotting factors due to mechanic stress and blood contact to foreign surfaces provokes hypercoagulability [3, 4]. Cannulation induces endothelial injury and ECMO induces blood flow disturbances, resulting in regional stasis to complete the risk profile. The increasing use of ECMO drives a progressive improvement of pumps and circuits, e.g., centrifugal pumps and heparin coating, reducing the incidence of thrombosis inside the device, seemingly allowing a reduction of anticoagulation [4].

Although bleeding and thromboembolic complications are feared complications during ECMO therapy and both contribute to mortality [5], the risk of venous thrombosis (VT) and thromboembolism (VTE) has just recently started gaining attention. Case reports and case series indicate that VT and VTE might influence outcome as well [6–10]. VTE were concurrently diagnosed in up to 10 % of cases [11] and in up to 18 % with routinely performed postdecannulation venous Doppler ultrasound [12]. Autopsy results of patients on veno-arterial ECMO support after cardiac surgery showed VT in 32 % of cases with a duration-dependent increase [13]. Considering the longstanding support of respiratory ECMO especially in patients that are bridged to lung transplantation [14], the true incidence of VT is probably still underestimated. This study aimed to evaluate the incidence of clinically diagnosed venous thrombosis and thromboembolism in patients treated with ECMO due to respiratory failure.

## Methods

### Study subjects

From a computerized database, we retrieved all patients who underwent high-flow ECMO at the pulmonary ICU at the University Hospital of Saarland (1.300 bed tertiary care hospital) from 04/2010 to 11/2015. This retrospective analysis includes all patients who survived ECMO therapy and could be discharged from ICU and autopsy findings of deceased patients treated in the same era. Patients that did not survive to discharge and for whom autopsy was refused are not analyzed.

Patients with preexisting thromboembolic disease such as acute pulmonary embolism or chronic thromboembolic pulmonary hypertension (CTEPH) and subjects with partial autopsy (e.g., selective autopsy of the thorax) were excluded from this analysis. Patients on pump-driven ECCO_2_R on 1/4-inch tubing were only included if the system was switched to high-flow ECMO on 3/8-inch. Four cases with a mean ECCO_2_R runtime of 10.7 ± 10.3 days were finally changed to full ECMO. The analysis was approved by the institutional review board (IRB; Ärztekammer des Saarlandes: #194/14 and #115/15). The necessity for informed consent was waived by the IRB due to the retrospective nature of the study.

### Extracorporeal membrane oxygenation/ECMO deployment/continuous veno-venous hemodiafiltration (CVVHD)

ECMO was initiated if PaO_2_/FiO_2_ (mmHg) was below 100 or if pH was below 7.20 for respiratory causes despite optimal conventional treatment. Patients had to have a potentially reversible cause of respiratory failure or the perspective of lung transplantation. Awake ECMO was initiated to prevent intubation in patients that were hypoxemic and/or hypercapnic (pH was below 7.20 or PaO_2_/FiO_2_ (mmHg) below 100) despite high F_i_O_2_. Twenty-nine patients had ECMO as a bridge to transplantation.

All patients were treated with veno-venous (vv) ECMO using mostly the femoral (draining) and jugular (return) veins as cannula entry sites. As a standard, we used 23 F draining cannulae at a length of 38 or 55 cm, as appropriate, and 19 F returning cannulae (Maquet, Rastatt, Germany) with a heparin coating. Eleven patients were cannulated with a bicaval double-lumen cannula (27 F or 31 F Avalon Elite, Avalon Laboratories, Rancho Dominguez, USA). Cannulation was done ultrasound-guided percutaneously by the staff intensivists. As standard oxygenator, a 7.0L-HLS or Quadrox-I primed with physiological saline solution on the Maquet CardioHelp platform was used. All tubings were 3/8 inch. ECMO circuits and oxygenators were visually checked for clots on a daily basis. Sedation was administered according to a protocol. Daily interruption of sedation was mandatory, except in hemodynamically unstable patients. The hemodynamic situation was monitored using an arterial and central venous line and pulmonary artery catheter when appropriate. Patients were weaned off vasopressors and sedation, whenever possible. Patients previously intubated and mechanically ventilated underwent tracheostomy, if extubation and awake ECMO were not possible.

CVVHD was done in all patients with regional citrate anticoagulation (Multifiltrate CiCa, Fresenius, Bad Homburg, Germany). The concentration of ionized calcium in the extracorporeal circuit is reduced to 0.25–0.35 mmol/L through chelation with citrate. Calcium is effectively eliminated during dialysis. To restore a physiological calcium level (normal range 2.2–2.6 mmol/L), calcium was added prior to return of the dialyzed blood to the patient’s circulation.

### Anticoagulation and transfusion

All patients were primarily treated with unfractionated heparin; in case of suspected heparin-induced thrombocytopenia (HIT), the regimen was switched to argatroban and HIT testing was performed. Patients that were HIT negative were switched back to heparin. The Extracorporeal Life Support Organization’s (ELSO) guidelines for anticoagulation were considered for anticoagulation management (http://www.elso.org/Resources/Guidelines.aspx). Additionally, we guided heparinization according to ECMO flow and aimed at >50 s for activated partial thrombin time at an ECMO flow ≥4.0 L/min. We aimed at higher aPTTs if ECMO flow was lower. We calculated the percentage of PTT above 50 s (number PTT values over 50 s divided by the total number of aPTT measurements times 100) to assess the effectiveness of anticoagulation and proportion of patients that were within the target range. In comparison with mean values of aPTT, this approach gives a more meaningful statement with regard to the quality of anticoagulation over a longer period of time.

Transfusion trigger for packed red blood cells (PRBC) according to our institutional protocol was central venous oxygen saturation (ScvO2) ≤65 % (despite optimal inotropic support if needed) and/or Hb ≤ 7.0 g/dL. Platelets were substituted if platelet count was below 20/µL or if diffuse bleeding occurred. Fresh frozen plasma (FFP) was substituted to optimize plasmatic coagulation and occasionally in situations of disseminated intravascular coagulation (DIC). Fibrinogen, PPSB (prothrombin-complex), factor XIII and ATIII were used as well according to the situation.

### Postmortem examination

We aimed for postmortem examination for all patients who died in our department. The autopsy was finally performed if the deceased or his relatives had agreed. The examination was performed following a standard protocol. Subjects receiving partial autopsy only were excluded from the analysis.

### Laboratory values

Routine laboratory values were determined directly prior to ECMO initiation and on a daily basis until cessation. Coagulation parameters including factors were determined only if bleeding was considered a problem. In cases of external cannulation and patient admission from external hospitals, laboratory values for the first days on ECMO were missing.

### Diagnosis of VT/VTE

Follow-up was done by analyzing all patients’ charts, available imaging data and autopsy results up to 1 year after ECMO therapy. Diagnosis was based on duplex sonography, CT scan or magnetic resonance angiography (MRA). In one case of clinically suspected pulmonary embolism after cannula removal, diagnosis without imaging was accepted. Screening data were complete for 81 % of patients.

### Statistical analysis

We described categorical data using frequencies and percentages. For continuous data, we used means and standard deviations as well as Kolmogorov–Smirnov test to check for normal distribution.

In bivariable analyses, we used Fisher’s exact test for categorical variables and Mann–Whitney *U* test or Student’s *t* test for continuous variables as appropriate. Variables with a *p* value of less than 0.1 in bivariable analyses were considered for a multivariable binary logistic regression model in order to estimate odds ratios and 95 % confidence intervals.

All statistical tests were two-sided, and *p* values of less than 0.5 were considered statistically significant. Statistical analyses were performed using IBM SPSS Statistics version 21 (SPSS Inc, Chicago, IL, USA).

## Results

### Patient characteristics

In total, data from 102 patients who underwent high-flow ECMO support were screened. Ninety-six patients were treated on the pulmonary ICU. Additionally, six patients treated on other ICUs on campus fulfilling the above-mentioned criteria and undergoing postmortem analysis were included. 44/96 patients (45.8 %) could be weaned from the device and discharged from ICU. 52/96 patients (54.1 %) died during extracorporeal support or within their ICU stay. Autopsy was refused in 29/52 cases (56 %).

We excluded all patients with preexisting VT/VTE and subjects with partial autopsy from the analysis. Two surviving patients, one had chronic thromboembolic pulmonary hypertension (CTEPH) and the other a thrombus in the right atrium on admission, as well as 8 autopsy cases were excluded.

**Fig. 1:**
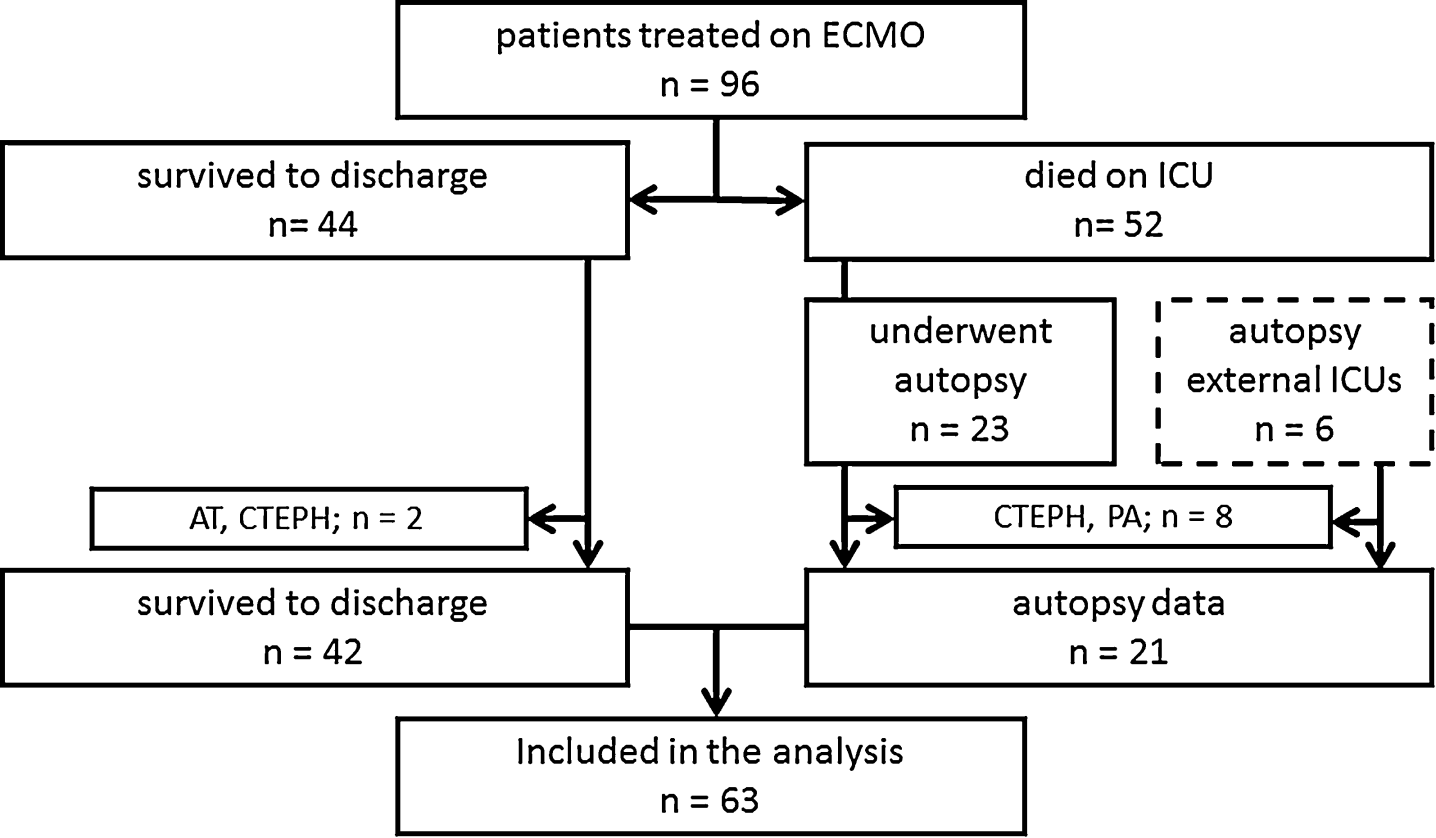
Consort diagram of patients included in the analysis. Six patients from the ICUs of the Depts. of Anaesthesiology and Cardiology were added to the cohort that met the inclusion criteria. *CTEPH* chronic thromboembolic pulmonary hypertension, *PA* partial autopsy only, *AT* preexisting atrial thrombus

Consequently, 63 patients treated from May 2010 to November 2015 were included in this retrospective analysis. Figure 1 shows the consort diagram for all subjects. The majority of patients were male [37/63 (59 %)], mean age was 46.0 ± 14.4 years, and mean time of ECMO support was 22.4 ± 17.0 days. Mean body mass index was 28.3 ± 9.8 kg/m^2^.

The main reason for ECMO initiation was bridge to recovery in acute respiratory distress syndrome (ARDS) in 34/63 patients (54 %). All, except one patient with cardiogenic shock, had pulmonary ARDS. The other patients (29/63; 46 %) had chronic or acute on chronic respiratory failure. In these patients, ECMO was initiated as a bridging procedure to lung transplantation. Underlying diseases were interstitial lung disease (14/29), cystic fibrosis (10/29), COPD (4/29) and pulmonary hypertension (1/29). In cases of external cannulation and patient admission from external hospitals, laboratory values for the first days on ECMO were frequently missing; this occurred in four patients concerning 5.6 ± 4.7 ECMO-days.

11 of 63 (17.5 %) patients were cannulated with a bicaval double-lumen cannula. Causes of death in the 21 patients undergoing postmortem examination were septic-toxic multiorgan failure in 17 (80.9 %) patients, two patients had acute right heart failure due to pulmonary embolism (9.5 %), one patient (4.8 %) died from cerebral edema and one patient (4.8 %) died from cerebral hemorrhage. Baseline characteristics are summarized in Table 1.

**Table 1:** Baseline patient characteristics of study patients

	All *N* = 63	No VT/VTE *N* = 34	VT/VTE *N* = 29	*p*
General characteristics				
Survivors	42 (66.7)	24 (70.6)	18 (62.1)	0.594
Male (%)	37 (58.7)	22 (59.5)	15 (40.5)	0.318
Age (years)	46.0 ± 14.4	47.6 ± 13.7	44.2 ± 15.3	0.465
Height (cm)	169.9 ± 9.7	168.8 ± 7.9	171.1 ± 11.6	0.451
Weight	70.9 ± 25.6	69.9 ± 25.2	72.1 ± 26.4	0.778
BMI	28.3 ± 9.8	27.7 ± 9.5	28.0 ± 10.3	0.390
CVVHD (%)	33 (52.4)	16 (47.1)	17 (58.6)	0.450
Time on ECMO (days)	22.4 ± 17.4	17.9 ± 13.9	27.7 ± 19.7	0.040
Cannulation				
VJI/VF	52 (82.5)	27 (79.4)	25 (86.2)	0.526
BCDL cannula	11 (17.5)	7 (20.6)	4 (13.8)	
Underlying disease				
ARDS (%)	34 (54.0)	19 (55.8)	15 (51.7)	
Pulmonary (%)	33 (97.0)	18 (52.9)	15 (51.7)	
Extrapulmonary (%)	1 (3)	1 (2.9)	0 (0)	
Bridge to LTX (%)	29 (46.0)	15 (44.1)	14 (48.3)	
CF (%)	10 (15.9)	6 (17.6)	4 (13.8)	
COPD (%)	4 (6.3)	2 (5.8)	2 (6.9)	
ILD (%)	14 (22.2)	7 (20.6)	7 (24.1)	
PH (%)	1 (1.6)	1 (2.9)	0 (0)	

*CVVHD* continuous veno-venous hemodialysis, *VJI/VF V.* jugularis interna/V. femoralis, *BCDL* bicaval double-lumen, *ARDS* acute respiratory distress syndrome, *LTX* lung transplantation, *ILD* interstitial lung disease, *COPD* chronic obstructive pulmonary disease, *PH* pulmonary hypertension, *CF* cystic fibrosis. Fisher’s exact test was used to examine differences between categorical variables, and numeric differences were analyzed using *t* test (Height) or Mann–Whitney *U* test as appropriate. Differences were considered as statistically significant if *p* ≤ 0.05. For multivariable test, values ≤0.1 were considered for further analyses

### VT/VTE incidence

Documented evaluation after clinical suspicion regarding VT/VTE was done in 51/63 patients (81.0 %), resulting in VT and VTE diagnosis in 29/63 (46.1 %) patients. In 24 of these 29 (83 %) patients, thrombosis was rated as cannula associated. Main thrombus localization was the inferior vena cava (IVC) in 15/29 (51.7 %) and internal jugular vein (IJV) in 14/29 cases (48.2 %). 8/29 patients (27.6 %) presented with thrombosis in more than one vein (Table 2).

**Table 2:** VT/VTE imaging modalities and thrombus localization

	*N* (%)
VT/VTE screening	51/63 (81.0)
VT diagnosis	29/63 (46.1)
Autopsy	11/29 (37.9)
CT angiography	6/29 (20.7)
Duplex	8/29 (27.6)
MRI	2/29 (6.9)
Other	2/29 (6.9)
VT/VTE	29/63 (46.0)
Thrombosis at cannula entry side	24/29 (82.8)
V. cava	15/29 (51.7)
V. jugularis interna	14/29 (48.2)
Pulmonary embolism	7/29 (24.3)
Multiple thrombosis sites	8/29 (27.6)

*VT/VTE* venous thrombosis/venous thromboembolism, *CT* computed tomography, *MRI* magnetic resonance imaging

Pulmonary embolism (PE) was diagnosed in 7/63 patients (11.1 %). PE was significantly more frequent in deceased patients than in survivors (*p* = 0.036). However, incidence of VT did not differ between these groups.

### Time on ECMO

Time on ECMO was statistically significant longer in patients diagnosed with VT/VTE than in patients without (*p* = 0.040). Mean ECMO time in patients with a thromboembolic event was 27.7 ± 19.7 days, compared to 17.9 ± 13.9 days in those patients without.

### Laboratory parameters, transfusion and substitution of coagulation factors

Analyzing mean laboratory values during ECMO therapy, we observed a significantly lower PTT (47.0 ± 12.3 vs. 53.6 ± 12.5 s; *p* = 0.037) in patients with diagnosed VT/VTE. The percentage of PTT values over 50 s was significantly reduced in patients with VT/VTE (31 ± 27 vs. 48 ± 27 %; *p* = 0.015).

In addition, VT/VTE patients had significantly lower fibrinogen levels (315.16 ± 77.97 vs. 406.60 ± 164.18 mg/dL; *p* = 0.026). Highly elevated D-dimers were seen in both groups of patients (*p* = 0.169). CRP-values were not different between groups (*p* = 0.679). Laboratory results are shown in Table 3. Platelet levels were not different between groups (*p* = 0.092). Mean hemoglobin levels were not different between groups; however, patients with VT/VTE received more transfusions of RPBC over time to achieve the same levels of hemoglobin, but the differences did not reach statistical significance (*p* = 0.068).

**Table 3:** Laboratory measurements in ECMO patients

	*N*	Ref.	All (*N* = 63)	No VT/VTE (*N* = 34)	VT/VTE (*N* = 29)	*p*
Hemoglobin (g/dl)	63	12–18	9.24 ± 0.73	9.25 ± 0.77	9.23 ± 0.96	0.994
Platelet count (platelets/µl)	63	140–400	143.09 ± 70.67	155.21 ± 85.50	128.88 ± 45.35	0.160
aPTT (s)	63	21–34	50.60 ± 12.75	53.63 ± 12.51	47.07 ± 12.30	0.037
%aPTT (s) > 50 s	63	n.a.	40.4 ± 28.5 %	48.35 ± 27.23 %	30.99 ± 27.41 %	0.015
INR	63	0.85–1.15	1.31 ± 0.50	1.30 ± 0.58	1.32 ± 0.39	0.148
Fibrinogen (mg/dl)	63	180–400	364.71 ± 138.5	406.60 ± 164.18	315.16 ± 77.97	0.026
C-reactive protein (mg/l)	63	0–5	120.77 ± 68.93	122.52 ± 78.85	118.73 ± 56.44	0.679
D-dimers (mg/l)	45	< 5	14.48 ± 7.79	12.68 ± 7.35	15.92 ± 7.98	0.169
Factor II (%)	35	70–120	84.57 ± 27.58	90.05 ± 25.66	80.45 ± 28.89	0.316
Factor V (%)	37	70–120	97.99 ± 25.44	104.48 ± 27.65	93.48 ± 22.64	0.155
Factor VII (%)	37	50–200	80.47 ± 22.36	80.64 ± 24.87	80.32 ± 20.65	0.965
Factor VIII:C (%)	38	70–120	152.32 ± 40.70	147.57 ± 46.92	156.89 ± 34.04	0.497
Factor IX (%)	45	70–120	100.20 ± 28.80	102.89 ± 31.62	97.40 ± 25.96	0.529
Factor X (%)	39	70–120	83.78 ± 21.66	83.52 ± 25.23	84.00 ± 18.72	0.728
Factor XI (%)	27	70–120	74.61 ± 24.35	71.03 ± 23.98	77.48 ± 25.09	0.505
Factor XII (%)	37	70–140	50.09 ± 22.30	45.31 ± 18.78	54.86 ± 25.12	0.376
Factor XIII (%)	51	70–140	66.22 ± 17.16	66.30 ± 18.19	66.14 ± 17.16	0.973

*aPTT* activated partial thromboplastin time, *INR* international normalized ratio, *%aPTT* denotes the number PTT values over 50 s divided by the total number of aPTT measurements times 100 (e.g., in the No VT/VTE group, patients had 48.35 % of measured values >50 s). Values are mean ± SD. Differences were analyzed using *t* test or Mann–Whitney *U* test (used for platelet count, aPTT, INR, fibrinogen, Factor X, XII, hemoglobin and CRP). Differences were considered as statistically significant if *p* ≤ 0.05

Transfusion of blood products and derivates are summarized in Table 4. PRBC and FFP were included in the multivariable analysis.

**Table 4:** Transfusion and substitution of coagulation factors (*N* = 63)

	All	NO VT/VTE	VT/VTE	*p*
PRBC Units (U)	21.86 ± 26.15	17.15 ± 22.54	27.38 ± 29.27	0.068
PRBC U/day	0.96 ± 1.12	0.88 ± 0.84	1.31 ± 1.88	0.629
Platelets U	5.00 ± 9.17	3.97 ± 7.68	6.21 ± 10.68	0.532
Platelets U/day	0.20 ± 0.45	0.18 ± 0.31	0.30 ± 0.68	0.862
FFP U	4.97 ± 10.36	3.12 ± 7.38	7.14 ± 12.83	0.092
FFP U/day	0.30 ± 0.87	0.24 ± 0.89	0.38 ± 0.85	0.138
AT III U**	1.61 ± 3.12	1.76 ± 3.67	1.43 ± 2.33	0.955
AT III U/day**	0.08 ± 0.17	0.09 ± 0.20	0.07 ± 0.13	0.861
Factor XIII	2.35 ± 3.38	1.71 ± 2.43	3.10 ± 4.16	0.118
Factor XIII U/day	0.12 ± 0.16	0.10 ± 0.14	0.14 ± 0.17	0.290

*PRBC* packed red blood cells, *FFP* fresh frozen plasma, *AT* *III* antithrombin III, *AT III* unit á 500 IE; Factor XIII unit á 1250 IE. Values are displayed as mean ± SD. Differences were analyzed using Mann–Whitney *U* test and were considered significant if *p* ≤ 0.05. For multivariable test, values ≤0.1 were considered for further analyses

When we compared different factors between the groups VT/VTE and no VT/VTE, time on ECMO,  %aPTT > 50 s, fibrinogen, amount of fresh frozen plasma (FFP) and packed red blood cells (PRBC) transfused yielded *p* values of less than 0.1 and hence were included in multivariable analyses. In a multivariable logistic regression analysis, time on ECMO and percentage of aPTT > 50 s were predictors of VT/VTE (Table 5).

**Table 5:** Multivariable analysis

	Odds ratio	95 % CI low	95 % CI high	*p*
%aPTT > 50 s	0.974	0.952	0.997	0.024
Time on ECMO	1.047	1.006	1.091	0.026
Fibrinogen	0.995	0.990	1.001	0.090
FFP	1.042	0.958	1.132	0.337
PRBC	0.984	0.950	1.019	0.361

*%aPTT* denotes the number PTT values over 50 s divided by the total number of aPTT measurements times 100, *FFP* fresh frozen plasma, *PRBC* packed red blood cells; Hosmer–Lemeshow test 0.56; Nagelkerkes *R* ^2^ 0.331

## Discussion

We set out to investigate the incidence and predictors of thrombosis and thromboembolism following ECMO therapy. Sixty-three patients, treated on ECMO in our center, were included in this analysis. The main findings of this retrospective analysis are: (1) at a rate of 46.1 %, thrombotic and thromboembolic events are frequent, (2) time on ECMO and level of anticoagulation influence the incidence of thrombotic and thromboembolic events and (3) these events tend to influence the outcome of these patients. Patient with a bicaval double-lumen cannula had no higher incidence of VT/VTE than other patients.

Critically ill patients are per se vulnerable for thrombotic and thromboembolic events. Kaplan et al. [15] published a VTE incidence of 37.2 % in ICU patients during severe sepsis and septic shock. Patients with viral ARDS are also at risk as suggested by data derived from the pandemic H1N1 [16]. Regarding non-infectious etiologies, pulmonary fibrosis is, for example, associated with a significantly elevated risk of thromboembolic disease [17].

Over and above these baseline risks, there are some certain characteristics for patients treated on ECMO. During extracorporeal assist, modification of blood composition as well as foreign surface and pump-mediated coagulopathy is one part of the problem. Cannulation is another. Starting with vascular injury at cannula entry site, the cannula splints the vessel until central areas are reached. This generates long areas of low flow and stasis along the cannula and thus provides an ideal location for thrombus formation. Possibly, additional indwelling catheters further obstruct the vessel. Taken together, these ICU patients are at high risk of VT/VTE.

However, there are still few data concerning the incidence of VT following ECMO and the aspect of VTE following respiratory ECMO therapy has not been investigated so far.

Cooper et al. [12] recently reported a retrospective analysis concerning the incidence of DVT in survivors of respiratory ECMO diagnosed by postdecannulation venous Doppler ultrasound. They used a 25 F multistage access cannula and a 23 F single-stage return cannula (Biomedicus, Mineapolis, MN) as standard cannulation configuration and a systemic anticoagulation with heparin aiming at a PTT of 1.5–2-times normal values. Surprisingly, DVT was found in merely 13/72 (18 %) of cases. This low incidence might be explained by a lack of reporting regarding V. cava thrombosis. Additionally, with a reported survival rate of 79 %, this cohort might be different from other cohorts.

We observed VT/VTE in 30/63 patients (47.6 %) that were mainly femoral/jugular cannulated. Due to the fact that thrombosis was mainly cannula associated, the IJV (46.7 %) and the IVC (50 %) were the most affected veins. A recent publication by Shafi et al. [8] reported upper-extremity deep vein thrombosis in 80 % of cases in a case series of 10 patients who were put on vvECMO with a dual-lumen cannula. In our present analysis, the rate of thrombosis associated with dual-lumen cannulas was not significantly different from other cannulation types and occurred in 4/11 cases (36.4 %).

To the best of our knowledge, this analysis is the first showing a high incidence of IVC thrombosis in ECMO patients. IVC thrombosis might account for roughly 2 % of lower limb deep vein thrombosis (DVT) [18]. IVC thrombosis harbors a 12–30 % risk of PE and a relevant risk of long-term complications such as chronic venous insufficiency, venous congestion and postthrombotic syndrome in up to 20 % of non-resolved thrombosis [18, 19]. PE as a major complication of VT was consequently diagnosed in 7/63 cases (11 %). We found one case of suspected PE directly following cannula removal and two cases of incidental PE diagnosed by CT scan. In most cases, VTs at cannulation sites are adherent to the venous wall and do not cause clinical symptoms. Up to now, there is no relevant literature on long-term complications following cannula-associated VT.

One of these patients died 24 days after weaning from device due to multiseptic organ failure following LTX and underwent postmortem examination.

The autopsy did not report VT or VTE possibly due to thrombus dissolution under prolonged anticoagulation. PE was consequently diagnosed in 5/21 deceased patients (24 %). Pulmonary embolism was only systematically evaluated for in deceased patients, so no adequate incidence for PE in the whole population can be given from this data. Reasons for PE in deceased patients might also be associated with circumstances and treatment modalities prior to end of life.

The management of anticoagulation, especially in prolonged ECMO support and in patients with sepsis and septic shock, is unclear. A recent ELSO survey showed that anticoagulation policies vary widely by center [20]. Bleeding is common in ECMO and cerebral bleeding, occurring at a rate of 4–15 %. In the majority of these patients, the outcome is deleterious [21–25]. Bleeding risk and transfusion requirements need to be balanced against VT/VTE risk, and this might likely end up in less anticoagulation. Mostly, aPTT around 50 s is recommended to minimize the risk of bleeding [4, 21, 22, 26]. This might provoke an increase in VT and VTE. We targeted a mean aPTT of 50–60 s, and our average aPTT was slightly above 50 s. The group of VT/VTE patients had a lower mean aPTT, possibly in line with the fact that they received more packed red blood cells. We assume that we missed our anticoagulation target due to bleeding complications. In fact, VT/VTE patients received more RPBC in total even though these differences did not reach statistical significance. Bleeding is often difficult to assess—a loss of 0.3 mL of blood per minute might be difficult to quantify but will lead to a loss of 480 mL per day. Additionally, there is no consensus on how to assess bleeding severity, especially if bleeding occurs at sites that are not easily accessible. Melena can be such an example; patients can lose significant amounts of blood, but quantification other than with the number of transfused RPBCs is impossible.

Our data are in line with the work of Rastan et al. [13] who analyzed autopsy results of patients undergoing veno-arterial ECMO support after cardiac surgery. They identified cardiac ECMO support > 2 days as an independent risk factor for systemic thromboembolic events and found systemic thromboembolic events in 36/78 patients (46.2 %) [13]. Compared to cardiac ECMO support, respiratory ECMO runs are often much longer. Cardiac ECMO runs were 3.5 ± 3.2 days in the study of Rastan et al. [13] versus 22.4 ± 17.4 in our pulmonary patients study. We also observed a trend toward longer duration of support in VT/VTE patients (*p* = 0.040). However, VT/VTE was also detected in short ECMO runs.

Our study has several limitations, which we would like to address. Many limitations are due to the retrospective nature and the low number of patients included in this work. One major limitation is based on the fact that we did not have a standardized screening protocol. The group of ECMO survivors has not been systematically analyzed. This might lead to an underestimation of the overall incidence.

Nevertheless, it has to be assumed that VT and VTE is an important and underdiagnosed complication of respiratory ECMO support and has impact on mid- and long-term outcomes. While we met common recommendations for anticoagulation on ECMO, the VT/VTE incidence was still high. Our results tend to underestimate the true incidence of VT/VTE in ECMO patients. Hence, it might be important to aim at higher aPTT times to prevent VT/VTE in a cohort of patients with a high-risk profile for VT/VTE.

## Conclusions

VT and VTE are common in patients following ECMO therapy. Rethinking anticoagulation might be needed as novel ECMO circuits show a high degree of biocompatibility and heparinization is to a lesser extent needed in the circuit as in the patient to prevent clotting. Current aPTT recommendations might be too low to avoid VT and VTE. The trend to recommend even lower aPTT targets might aggravate the problem in the future. Higher aPTT recommendations have to be carefully weighed against the risk of bleeding, as bleeding, especially intracranial hemorrhage, has deleterious consequences.

 To achieve optimal outcomes, we recommend VT screening following ECMO therapy.
